# Action of Putrid Material on the Animal Organism

**Published:** 1868

**Authors:** 


### ACTION OF PUTRID MATERIAL ON THE ANIMAL ORGANISM.

The following conclusions have been derived by Dr. Moriz Hemmer, of Munich, from his researches on the nature and action of putrid fluids:

1. Putrid infection causes severe acute inflammation in the intestinal mucous membrane and the glands of the chylopoietic system. 2. It excites very violent central irritation. 3. By it the blood is changed into a dark colored, thin, and scarcely coagulable fluid. 4. It causes the rapid approach of putrefaction. 5. The putrid poison is an albuminoid body undergoing change, not fluid or gaseous, but solid. 6. The poison acts in imperceptibly small doses; and, with regard to its intensity, can be compared only with the most active toxic agents known to us—some vegetable alkaloids, curare, the snake poison, etc. 7. It is insoluble in absolute alcohol, soluble in water. 8. It resists a heat of 100 degrees centigrade. 9. It acts as a ferment, and induces zymotic changes in the blood. 10. The action of the putrid poison is exerted on the albuminoid materials of the plasma of the blood. 11. An analogy may generally be recognized between putrid infection and the infectious diseases. 12. The morbid material of the infectious diseases are, therefore, putrid poisons, and possess the properties of the same. 13. The varying action of the morbid materials in the infectious diseases depends upon a special modification of the putrid poison.—*British Medical Journal, from Blatter f. Staats-Arzneikunde.*

